# Proteolytic Resistance Determines Albumin Nanoparticle Drug Delivery Properties and Increases Cathepsin B, D, and G Expression

**DOI:** 10.3390/ijms241210245

**Published:** 2023-06-16

**Authors:** Ekaterina P. Kolesova, Vera S. Egorova, Anastasiia O. Syrocheva, Anastasiia S. Frolova, Dmitry Kostyushev, Anastasiia Kostyusheva, Sergey Brezgin, Daria B. Trushina, Landysh Fatkhutdinova, Mikhail Zyuzin, Polina A. Demina, Evgeny V. Khaydukov, Andrey A. Zamyatnin, Alessandro Parodi

**Affiliations:** 1 Scientific Center for Translation Medicine, Sirius University of Science and Technology, 354340 Sochi, Russia; e.p.kolesova@gmail.com (E.P.K.); egorova.vs@talantiuspeh.ru (V.S.E.); syrocheva.ao@talantiuspeh.ru (A.O.S.); frolanasta@gmail.com (A.S.F.); dkostushev@gmail.com (D.K.); kostyusheva_ap@mail.ru (A.K.); seegez@mail.ru (S.B.); khaydukov@mail.ru (E.V.K.); 2 Institute of Molecular Medicine, Sechenov First Moscow State Medical University, 119991 Moscow, Russia; 3 Martsinovsky Institute of Medical Parasitology, Tropical and Vector-Borne Diseases, Sechenov First Moscow State Medical University, 119991 Moscow, Russia; 4 Department of Biomedical Engineering, Sechenov First Moscow State Medical University, 119991 Moscow, Russia; trushina.d@mail.ru; 5 Federal Scientific Research Center “Crystallography and Photonics”, Russian Academy of Sciences, 119333 Moscow, Russia; polidemina1207@yandex.ru; 6 School of Physics, ITMO University, Lomonosova 9, 191002 St. Petersburg, Russia; l.fatkhutdinova@metalab.ifmo.ru (L.F.); mikhail.zyuzin@metalab.ifmo.ru (M.Z.); 7 Shemyakin-Ovchinnikov Institute of Bioorganic Chemistry, Russian Academy of Sciences, 117997 Moscow, Russia; 8 Belozersky Institute of Physico-Chemical Biology, Lomonosov Moscow State University, 119992 Moscow, Russia; 9 Faculty of Health and Medical Sciences, University of Surrey, Guildford GU2 7X, UK

**Keywords:** cathepsins, albumin nanoparticles, biological carriers, drug delivery, lysosomal enzymes, protein degradation

## Abstract

Proteolytic activity is pivotal in maintaining cell homeostasis and function. In pathological conditions such as cancer, it covers a key role in tumor cell viability, spreading to distant organs, and response to the treatment. Endosomes represent one of the major sites of cellular proteolytic activity and very often represent the final destination of internalized nanoformulations. However, little information about nanoparticle impact on the biology of these organelles is available even though they represent the major location of drug release. In this work, we generated albumin nanoparticles with a different resistance to proteolysis by finely tuning the amount of cross-linker used to stabilize the carriers. After careful characterization of the particles and measurement of their degradation in proteolytic conditions, we determined a relationship between their sensitivity to proteases and their drug delivery properties. These phenomena were characterized by an overall increase in the expression of cathepsin proteases regardless of the different sensitivity of the particles to proteolytic degradation.

## 1. Introduction

**Figure 1:**
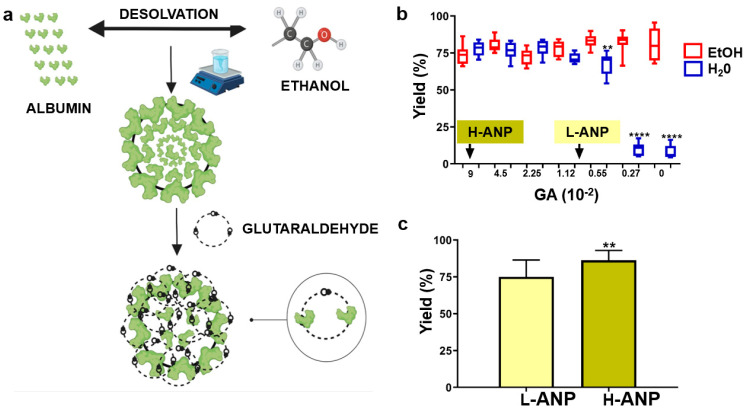
Determination of glutaraldehyde concentration to generate H−ANPs and L−ANPs: (**a**) ANP synthesis schematic. (**b**) Nanoparticle recovery after ANP cross-linking with different concentrations of glutaraldehyde and further resuspension in ethanol (red) or water (blue). In consideration of these data, H−ANPs and L−ANPs were synthesized with 0.09% and 0.009% of cross-linker, respectively. Significance was calculated via Student’s *t*-test: **** = *p* < 0.0001 (**c**) L-ANP and H-ANP average synthesis yield. Data represent mean ± SD. Significance was calculated via Student’s *t*-test: ** = *p* < 0.005.

Cellular proteases regulate important cell functions and guarantee proper protein degradation and amino acid recycling [1]. Their action is mostly (but not solely) concentrated in lysosomal organelles [2], where together with other hydrolases, they degrade aged cellular biological molecules and foreign elements [3]. One of the most investigated families of cellular proteases is represented by lysosomal cathepsins, which are divided into cysteine, serine, and aspartic cathepsins depending on the amino acid sequence present in their active site [2]. Despite their role in cell homeostasis, cathepsins also have important functions in pathologic conditions [4]. In cancer, they were recently shown to modulate tumor cell phenotype [5], aggressiveness [6], ability to spread in distant organs [7], and metabolism [8]. Recent investigations showed that the activity of these enzymes can determine the efficiency of anticancer treatments [9,10] by favoring or counteracting the apoptotic process. However, in this context, the relationship between lysosomes, their enzymes, and new pharmaceutical formulations was often overlooked [11]. Most of the current nanoformulations are sequestered in the endosomal compartment [12], where the payload is released. Over the last three decades, new advances in material science have highlighted the possibility of manipulating many materials at the nanoscale, including molecules of inorganic, organic, and biological natures [13]. Between them, protein nanocarriers represented a breakthrough in pharmaceutical development with albumin serving as an ideal biological material to generate nanoparticles [14], thanks to its abundance, biocompatibility, natural ability to transport drugs, and cost-effectiveness. Different protocols were developed to fabricate albumin nanoparticles (ANPs) [15], and one of the most promising in terms of reproducibility is desolvation followed by cross-linking [16]. In this synthetic route, ethanol is used to denature albumin molecules, inducing their self-assembly in nanoclusters, eventually stabilized with a chemical cross-linker such as glutaraldehyde (Figure 1a). Glutaraldehyde is a common cross-linker for albumin nanoparticle synthesis [17,18], leading to the attainment of particles with excellent stability, low polydispersity, and uniform size and shape. Even though the quest for more biocompatible cross-linkers remains paramount [19,20], glutaraldehyde is perfectly suited for this goal since it can affect particle biodegradation and it has been widely used in nanocarrier synthesis [21,22,23]. It can affect particle biodegradation [24]. In this work, we further explored this concept by investigating the effect of different glutaraldehyde concentrations on increasing ANP proteolytic resistance and exploring these results in terms of drug delivery properties. Finally, we evaluated the effects of the particle proteolytic resistance on the expression of proteases representative of cysteine, serine, and aspartic cathepsins.

## 2. Results

### 2.1. Determination of Cross-Linker Concentrations to Fabricate Lightly (L−ANP) and Heavily (H−ANP) Cross-Linked ANPs

ANPs were synthesized via protein desolvation in ethanol, followed by cross-linking with glutaraldehyde (Figure 1a). To test the cross-linker ability in increasing particle resistance to proteolysis, we generated lightly (L−) and heavily (H−) cross-linked ANPs using different amounts of glutaraldehyde. For this reason, we first determined the minimal concentration of the cross-linker effective for generating a stable product with a synthetic yield of at least higher than 60%. Various nanoparticle syntheses were established with increasing concentrations of glutaraldehyde, as described in the Materials and Methods section. A significant drop in ANP yield was observed at 0.0055% of glutaraldehyde. Below this concentration, the recovery of nanoparticles was negligible (Figure 1b). In consideration of this analysis, we chose to fabricate nanoparticles with 0.009% and 0.09% of cross-linker to generate L-ANPs and H-ANPs, respectively. Then, we tested the reproducibility of these protocols, determining that H-ANP synthetic yield was significantly higher (87%) than L-ANP (75%) (Figure 1c).

### 2.2. Analysis of Particle Physical and Surface Properties

**Figure 2:**
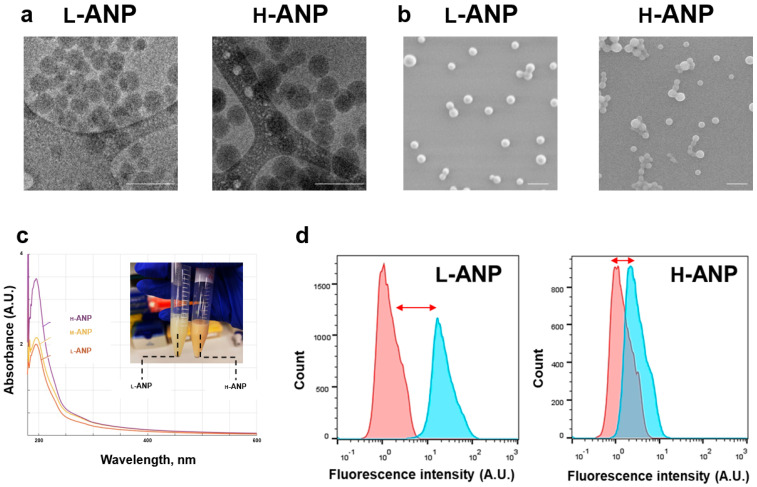
Evaluation of particle physical and surface properties: (**a**) Investigation of particle size via cryo-TEM microscopy analysis; scale bars correspond to 200 nm. (**b**) Particle SEM analysis; scale bars correspond to 200 nm. (**c**) U.V./Vis spectrum of H−ANPs, L−ANPs, and mildly cross-linked albumin nanoparticles (M-ANPs). In the inset, L−ANP and H−ANP solutions are shown. (**d**) Fluorescent intensity shift of unmodified nanoparticles (red curve) after covalent binding of FITC-NHS (blue curve).

Nanoparticle size was measured through different methods. Cryo-TEM (Figure 2a) and SEM (Figure 2b) showed a similar spherical shape and a size between 50 and 120 nm in both the formulations. A higher electron density and moderate particle aggregation for H-ANPs was detected, probably because of the higher concentration of glutaraldehyde used. Compared to SEM measurement (Figure S1a), size increased considerably when measured via dynamic light scattering in both the samples (155.5 nm for L-ANPs and 228.5 for H-ANPs) (Figure S1b), in particular for H-ANPs. The surface charge of the nanoparticles was measured via Z-Pot analysis in phosphate buffer at physiological pH (7.2). The surface charge of both the nanoparticles was negative, with H-ANPs being considerably more negative than L-ANPs (−38.8 vs. −28.4) (inset Figure S1). After the synthesis, the particles were also characterized by a different color shade (inset Figure 2c). Light absorbance was measured via UV-Vis spectroscopy analysis, and for this experiment we also introduced a new batch of particles generated with a mild concentration of cross-linker (M-ANP). All the particles showed a similar absorbance profile with a peak close to 200 nm, and H-ANPs showed the highest absorbance, while L-ANPs showed the lowest. To determine the glutaraldehyde saturation degree of free amines, we tested particle modification ability with a fluorescent dye using NHS chemistry (which covalently binds the fluorophore to amino groups). After the chemical modification and washing, particle fluorescence was analyzed through flow cytometry. Compared to unmodified nanoparticles, L-ANPs showed a large shift in the fluorescence axis, while the H-ANP histogram barely moved, demonstrating that the concentration of glutaraldehyde used was almost effective in saturating all the amine groups on the surface of the particles (Figure 2d).

### 2.3. Particle Loading and Release in Normal and Proteolytic Conditions

**Figure 3:**
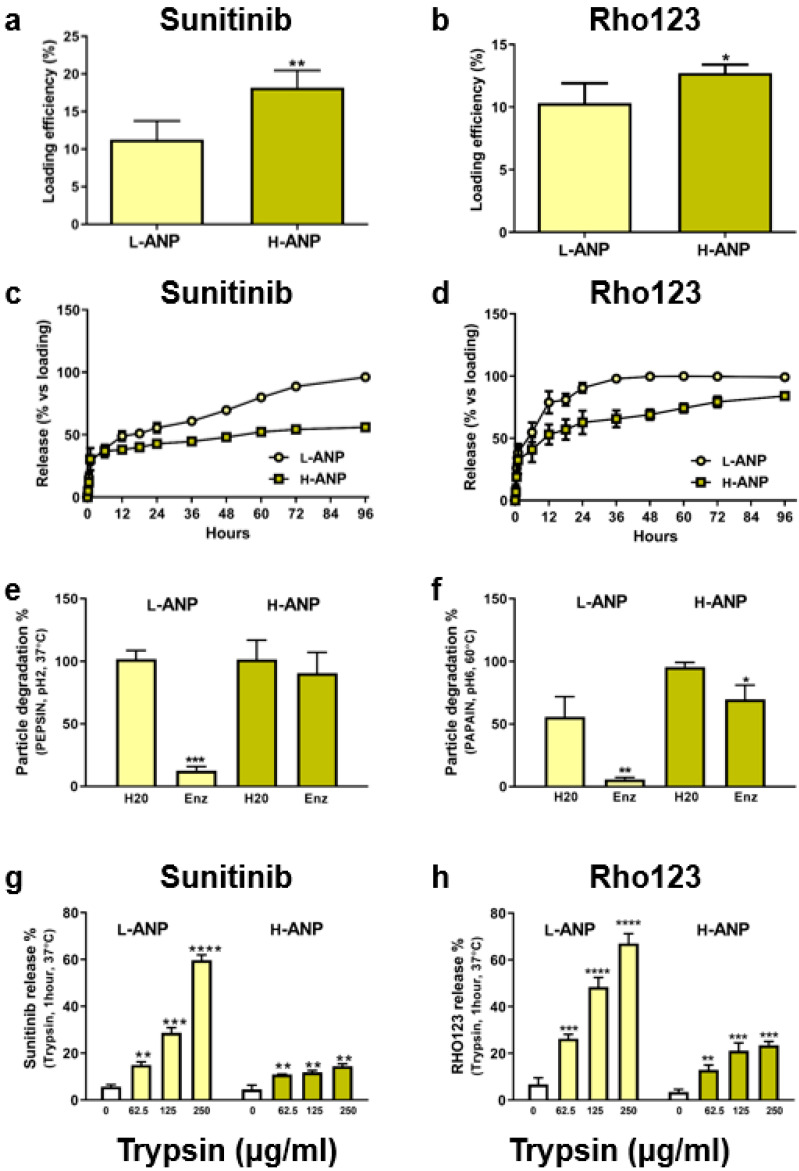
Loading and release properties in normal and proteolytic conditions: (**a**) L-ANP and H-ANP loading efficiency with Sunitinib and (**b**) Rho123. (**c**) L-ANP and H-ANP release profile of Sunitinib and (**d**) Rho123. Data represent mean ± SD. Significance was calculated via Student’s *t*-test. (**e**) Nanoparticle degradation after incubation with pepsin and (**f**) papain. (**g**) Release of Sunitinib and (**h**) the fluorescent dye Rho123 after particle incubation in trypsin. Data represent mean ± SD. Significance was calculated via one-way ANOVA followed by Dunnett’s test: * = *p* < 0.05, ** = *p* < 0.005, *** = *p* < 0.0005, **** = *p* < 0.0001.

To evaluate the effect of the cross-linker on drug loading and release, we selected two molecules of therapeutic and imaging interest which were poorly soluble in water: Sunitinib malate and Rhodamine 123, respectively. Sunitinib is a tyrosine kinase inhibitor used as a chemotherapeutic against different cancers, including breast and renal cancer cells [25,26]. Rhodamine 123 is a viable dye used to stain cell mitochondria [27]. Both the nanoparticles were loaded after synthesis by incubating 10 mg of carriers in 50 µL of dye dissolved in DMSO with mild agitation overnight. After many washes, the loading yield was calculated via the direct method by incubating the nanoparticles at 37 °C in DMSO as described in the Materials and Methods section. In both cases, loading yield was higher for H-ANPs compared to L-ANPs. In particular, Sunitinib loading yield was 18.2% in H-ANPs vs. 11.2 in L-ANPs, while Rhodamine 123 loading yield was 12.72% in H-ANPs vs. 10.31% in L-ANPs (Figure 3a,b). These molecules also showed similar release kinetics, with H-ANPs characterized by a slower release than L-ANPs. At the end of the experiment, similar to the loading calculation, the highest difference between the two formulations was registered for Sunitinib (Figure 3c,d).

Particles’ resistance to a proteolytic environment was tested through different commercial enzymes, dissolved in appropriate buffer pH and temperature. We tested pepsin (pH 2; 37 °C), papain (pH 6; 60 °C), commercial protease (pH 8; room temperature), and cell-grade trypsin (physiological pH; 37 °C). Compared to non-proteolytic conditions, when incubated with pepsin, L-ANP degradation was significantly higher compared to H-ANPs, which showed only a small decrease in weight (Figure 3e). Interestingly, in the same experimental setting, after labeling albumin molecules with AF555 via NHS chemistry, the degradation of L-ANPs decreased (Figure S2a). Similar data were obtained when the particles were incubated with papain. Here, we also registered an increase in L-ANP degradation in non-proteolytic conditions, showing that the cross-linker can increase particle thermic resistance (Figure 3f). A higher degradation of L-ANPs in non-proteolytic conditions was also observed after incubation of the particles with the buffer of commercial protease (pH 8), while H-ANP recovery was slightly reduced; however, in both cases, particle degradation increased in proteolytic conditions (Figure S2b). These data show that the cross-linker also induced a certain resistance against basic pH. Trypsin was used to evaluate Sunitinib and Rhodamine 123 release in proteolytic conditions. The drug release depended on trypsin concentration and L-ANPs showed a significantly higher release of both the molecules compared to H-ANPs (Figure 3g,h).

### 2.4. Determination of Intracellular Drug Release

**Figure 4:**
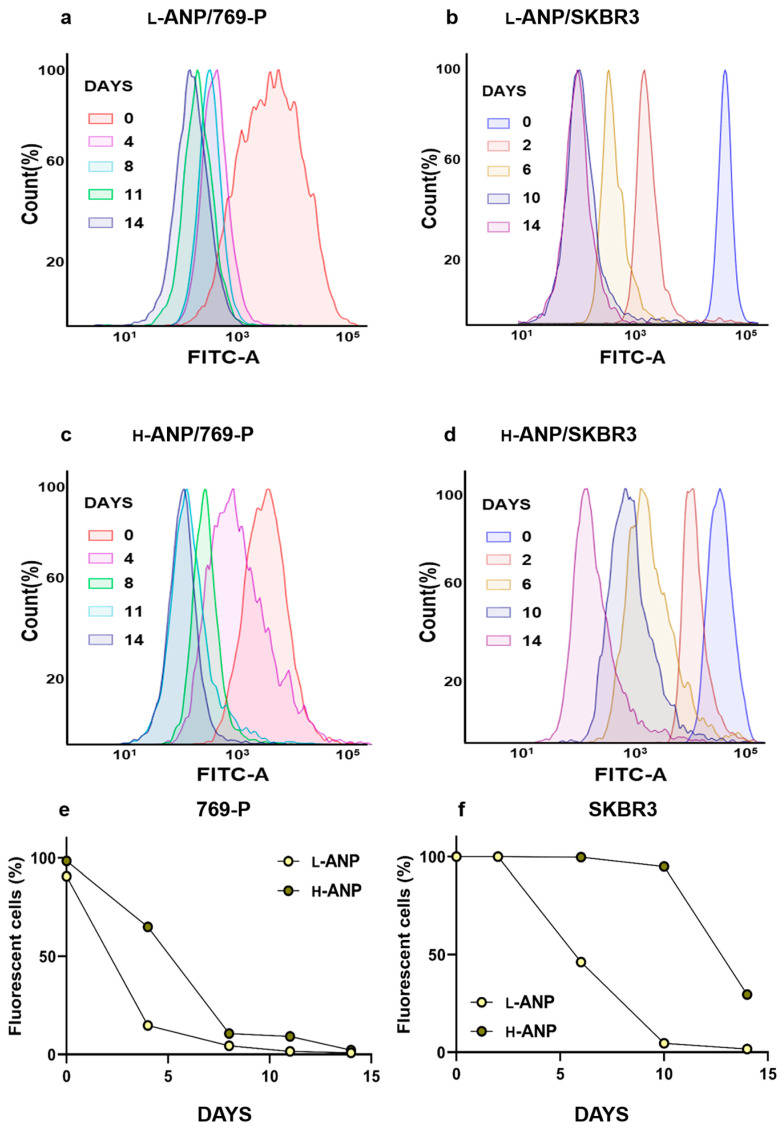
Intracellular release of Rho123 and drug delivery properties: (**a**) Flow cytometry overlay histograms for FITC-A channel demonstrating percentage of fluorescent 769-P and (**b**) SKBR3 cells after treatment with Rho123-loaded L-ANPs over time. (**c**) Flow cytometry overlay histograms for FITC-A channel demonstrating percentage of fluorescent 769-P and (**d**) SKBR3 cells after treatment with Rho123-loaded H-ANPs over time. (**e**) Decrease of the percentage of fluorescent cells in 769-P and (**f**) SKBR3 populations.

**Figure 5:**
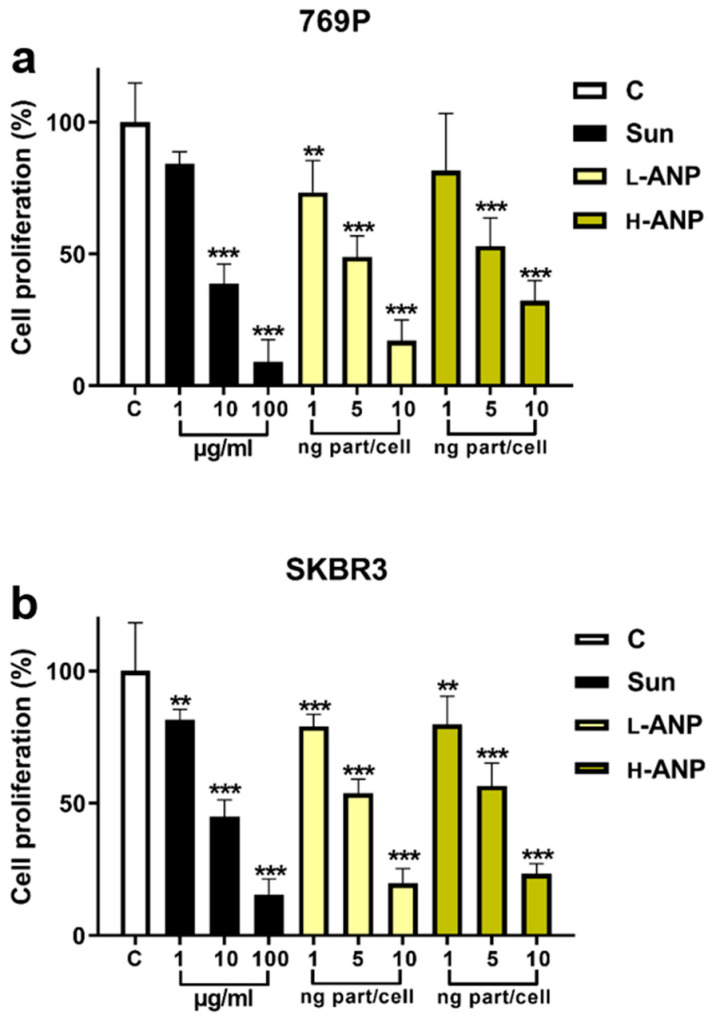
Sunitinib-loaded ANP toxicity: (**a**) Determination of 769-P and (**b**) SKBR3 viability 48 h after overnight incubation with increasing doses of free drug and Sunitinib-loaded ANPs. Significance was calculated via one-way ANOVA followed by Dunnett’s test: ** = *p* < 0.005, *** = *p* < 0.0005.

Before treating human breast cancer SKBR3 cells and human renal cancer 769-P cells, we evaluated the particle impact on cell viability. The treatment was performed with increasing concentrations of nanoparticles for 72 h. No significant cell mortality was registered, except at the highest concentration of H-ANP in 769-P, in which we detected a non-significant decrease (Figure S3). Further experiments were all performed for 6 h or overnight at a maximum concentration of 10 ng/cell. To evaluate any potential difference in intracellular drug release between L-ANPs and H-ANPs, the nanoparticles were loaded non-covalently with Rhodamine 123 and administered to the cells. After 6 h, the cells were washed, and the fluorescence was measured at different time points within a time window of 14 days via flow cytometry. In both the cell lines used, we observed a faster decrease of the fluorescence with L-ANPs (Figure 4a–f). Finally, we investigated the cytostatic properties of Sunitinib-loaded nanoparticles. First, we measured the sensitivity of the cells for Sunitinib, demonstrating that this molecule could decrease the cell viability in a concentration-dependent way (Figure 5a,b). The treatment was performed overnight with increasing doses of drug and then the medium was replaced, and cell viability was evaluated after 48 h. Nanoparticle treatment was performed in a similar fashion, and in this case we also observed a dose-dependent toxicity after cell treatment with Sunitinib-loaded L-ANPs and H-ANPs. Interestingly, despite a lower loading yield, L-ANPs showed a higher cytotoxic power than H-ANPs (Figure 5a,b).

### 2.5. Determination of Lysosomal Cathepsin B, D, and G Expression

**Figure 6:**
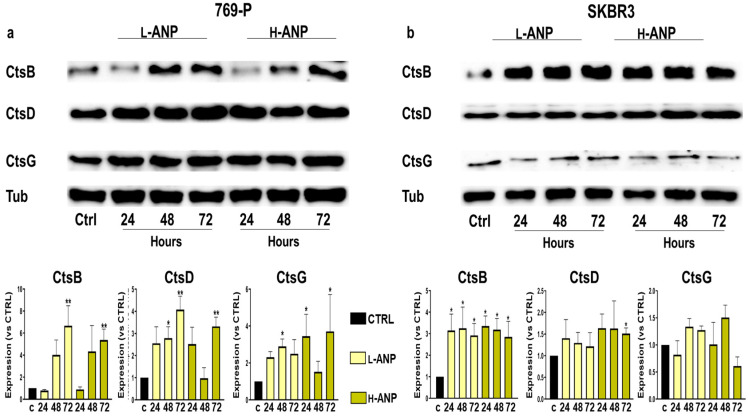
Effect of ANPs on cathepsin B, D, and G expression: (**a**) Protein expression of CtsB, CtsD, and CtsG, and quantification after normalization with Tubulin after 769-P; and (**b**) SKBR3 treatment with ANP for 6 h. The samples were collected 24, 48, and 72 h after the treatment. Data represent mean ± SD. S Significance was calculated via one-way ANOVA followed by Dunnett’s test: * = *p* < 0.05, ** = *p* < 0.005.

To determine potential changes in the lysosomal enzymatic composition of 769-P and SKBR3 after nanoparticle internalization, we treated the cells for 6 h, and after extensive washing, the cells were collected at 24, 48, and 72 h after the treatment. We determined the protein expression of Cathepsin (Cts) B, D and G, representative of the cysteine, serine, and aspartic cathepsin sub-families. A general evaluation of the data showed that, after L-ANP or H-ANP treatment, both the cell lines increased the expression of these proteins similarly in comparison with untreated cells (Figure 6a,b).

In both the cell lines, CtsB was the protein that increased the most: it showed a time-dependent increase in 769-P, while in SKBR3 the protein level augmented consistently at all the time points considered. CtsD expression showed a smaller increase compared to CtsB both in 769-P and SKBR3 cells. Interestingly, after 769-P treatment with H-ANPs, an increase of CtsD expression was detected at 24 and 72 h, while at 48 h the level of the protein was similar to the control. CtsG also showed a small general increase in 769-P with both the particles and at all the time points, while slight changes were observed in SKBR3, where at 72 h a non-significant decrease compared to the control cells could be observed.

## 3. Discussion

The generation of new pharmaceutical formulations exploiting nanocarriers has paved the way for developing novel targeted drug delivery approaches [28,29]. In this context, drug release kinetics is a fundamental step to achieving the generation of an effective treatment, and, in the absence of external triggers, particle degradation was shown to represent the most crucial phenomenon to control therapeutic release [30,31]. Particle degradation depends on particle material [32], manipulation [33], and biocompatibility, representing its ability to degrade in a biological environment, and in particular in the lysosomes, since they represent the most common final destination of internalized particles. With biological nanoparticles and in particular with protein carriers, lysosomal activity is often taken for granted as the cellular mechanism at the basis of particle degradation after internalization [34,35,36]. However, a more thorough analysis needs to be performed when nanoparticles are stabilized via cross-linkers, as shown by investigations focused on understanding the potential of ANPs generated via desolvation/cross-linking methods for gene delivery [37]. In this work, particle stabilization was achieved with glutaraldehyde that was previously shown to impart nanoparticles with resistance against proteases [24], potentially affecting their drug delivery properties and lysosomal biology. For this reason, we first determined the minimal amount of cross-linker necessary to stabilize the particles in aqueous media and eventually we chose two different concentrations of glutaraldehyde with an order-of-ten difference, generating L-ANPs and H-ANPs. While both these protocols allowed for a good particle synthetic yield as a function of the initial albumin concentration, it was not surprising that we consistently detected a higher carrier recovery for H-ANP, considering the stabilizing effect of the glutaraldehyde. Further analysis of the nanoparticle size showed a similar shape and size distribution of L-ANPs and H-ANPs evaluated with SEM and cryo-TEM, confirming previous works testing this platform [16]. These analyses showed that the average diameter of the nanoparticles was around 90 nm, underestimating the DLS measurement. These discrepancies were also shown for other delivery platforms [38,39]. The calculation of the hydrodynamic diameter from a diffusion measurement involves significant assumptions, including the core and the surface adsorption layer.

This analysis also highlighted an increase of H-ANP PDI that can be related to the higher cross-linking degree clustering few particles together. More importantly, the glutaraldehyde showed a significant impact on nanoparticle surface charge at physiological pH. Despite a net color change because of a differential light absorbance, both the groups of particles showed a negative surface charge, with H-ANP consistently lower than L-ANP. This effect was probably because of the saturation of the amine groups by glutaraldehyde, as confirmed by the binding efficiency of the fluorescent label via NHS chemistry.

**Figure 7:**
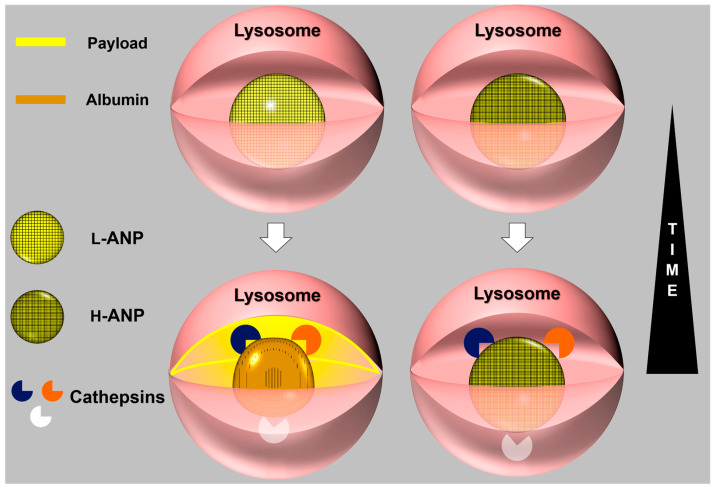
Major outcomes of the work: Different concentrations of cross-linker change the chemical–physical properties of the particles despite decreasing their surface charge and saturating free amino groups. More importantly, the cross-linker increased drug loading efficiency, retention, and resistance to proteolytic activity. As a result of these properties, L-ANPs showed a higher cytostatic power than H-ANPs despite a lower drug content. However, H-ANP and L-ANP internalization similarly increased the cellular content of Cts B, D, and G, demonstrating that resistance to proteolysis does not affect the expression of these enzymes.

Sunitinib and Rhodamine 123 were chosen as the models of payload. These fluorescent molecules were non-covalently linked to the particles to investigate their release as potential therapeutics. Both these molecules are hydrophobic and their loading showed higher results in H-ANPs than L-ANPs, in particular with Sunitinib. We can speculate that this phenomenon depended on the occurrence of hydrophobic interactions, even though more evidence should be collected on the effect of the cross-linker on particle porosity. In DMSO, drug release was consistently slower for H-ANPs than L-ANPs, particularly with Sunitinib. However, investigation on a larger portfolio of molecules is necessary to understand the ability of the particles to accommodate a payload as a function of the amount of glutaraldehyde used. Nonetheless, this evidence provides initial proof that the cross-linker can affect ANP delivery properties. Additionally, the cross-linker imparted a higher resistance to chemical- and temperature-dependent degradation. Our data show that free amines in the L-ANPs might play a role in favoring particle proteolysis (pepsin), since the degradation of these carriers was mitigated when the amine groups were linked to a fluorescent molecule via NHS. However, more investigations are needed to verify the specific role of the fluorophore in decreasing carriers’ degradation. When tested with trypsin, L-ANPs loaded with Sunitinib or Rhodamine 123 demonstrated a burst release of the payloads compared to H-ANPs, demonstrating that the sensitivity of these particles to proteolytic activity can determine their drug delivery properties. Further experiments were performed to evaluate intracellular drug release. For this purpose, we verified that, in our experimental conditions, no significant particle toxicity occurred confirming other evidence reported for this kind of delivery platform [40]. Glutaraldehyde has a known toxicity profile on living cells [41]; however, it exerts it mostly during the cross-linking reaction and therefore it was mitigated by extensive washing steps performed prior to cell administration. We chose two cancer cell lines investigated for lysosomal protease (cathepsin) expression: 769-P human renal cancer cells [42] and SKBR3 human breast cancer cells [10]. Both the cell lines were treated with ANP loaded with Rhodamine 123, and we measured the decrease of the dye fluorescence within 2 weeks. In both the cell lines, the signal derived from L-ANPs disappeared faster than H-ANPs. Despite cell proliferation that decreases nanoparticle content at each division [43], the fluorescence decrease could be explained by a slower release of this molecule from H-ANPs, but also because of the faster degradation of L-ANPs. In line with the data obtained with the fluorescent dye, we evaluated the effects of Sunitininb-loaded nanoparticles on the cell viability. Both L-ANPs and H-ANPs showed a significant dose-dependent ability in decreasing cell viability 48 h after treatment. However, despite their lower loading properties, L-ANPs showed similar or higher cytostatic properties. We did not detect a significant difference in cathepsin content, despite an overall expression increase detected both after L-ANP and H-ANP treatment. Within all the cathepsins tested, CtsB showed the higher increase, and this enzyme was shown to react to different stimuli [2,44,45] and to be effective in degrading ANPs [24], even though to our knowledge no kinetic expression investigation has ever been performed. Interestingly, it was reported that CtsB has a slight effect on mature albumin in comparison with other cystein cathepsins [46] and CtsD [47]. In this scenario, future investigations on the expression of more enzymes and their natural inhibitors [42] could shed light on the specific activity of different cathepsins in degrading ANPs. The major outcomes in this work are shown in Figure 7.

## 4. Materials and Methods

### 4.1. Chemicals and Reagents

The cell lines derived from human renal cancer, 769-P, and human breast cancer SKBR3 were purchased from American Type Culture Collection (Manassas, VA, USA). The cells were grown in RPMI 1640, supplemented with 10% fetal bovine serum and a 1% mixture of penicillin–streptomycin antibiotics (all from Gibco, Waltham, MA, USA) at 5% CO_2_ and 37 °C in a humidified atmosphere containing 5% CO_2_. Cell lines were authenticated with STR DNA Profiling Analysis (GORDIZ, Moscow, Russia). Cell lines were checked with the MycoAlertTM Mycoplasma Detection Kit (Lonza, Basel, Switzerland) and were free of contamination. Bovine serum albumin, ethanol, pepsin, and commercial protease were purchased from Sigma-Aldrich (Saint Louis, MO, USA), while glutaraldehyde was purchased from PanReac AppliChem (Barselona, Spain). Cellular-grade trypsin was purchased from Gibco, while papain was purified in our lab. All the reagents necessary to perform Western blot were purchased from Biorad (Hercules, CA, USA), while the antibodies were purchased from Abcam (Cambridge, UK). Lysosensor green was purchased from Invitrogen (Waltham, MA, USA).

### 4.2. Nanoparticle Synthesis and Determination of Glutaraldehyde Concentrations to Generate Lightly Cross-Linked ANPs (L-ANPs) and Heavily Cross-Linked ANPs (H-ANPs)

Albumin nanoparticles were synthesized using desolvation followed by cross-linking [15]. Protein denaturation and nanoparticle formation occurred by adding dropwise ethanol at room temperature to a water solution of bovine serum albumin (20 mg/mL) in a ratio of ethanol/water 4 to 1. The addition of ethanol was carried out at constant stirring and speed (2 mL/min) using a digital pump system (Masterflex L/S). Then, glutaraldehyde (25% solution) dissolved in 1 mL of ethanol was added dropwise as well. The solution was stirred overnight at room temperature.

To evaluate the minimal amount of glutaraldehyde necessary to stabilize the particles, different amounts of cross-linker were added. On the next day, the particles of each batch were divided into equal amounts and centrifuged at 13,000× *g*, the supernatant was discarded, and the particles were resuspended in ethanol or water and kept at room temperature for 24 h. All the 1.5 mL tubes used in this experiment were previously weighed. Then, the vials were centrifuged at 13,000× *g*, the supernatant was discarded, and the vials were dried at 65 °C overnight. On the next day, the vials were weighed again and the colloidal stability was calculated as a function of the loss in weight between nanoparticle resuspended in ethanol and in water as shown in the graph. For the further experiments, glutaraldehyde was used at a final concentration of 0.009% for L-ANPs and 0.09% for H-ANPs. Particles were eventually washed via centrifugation many times and resuspended in water before determining their final concentration.

### 4.3. Nanoparticle Size and Surface Charge Characterization

The ζ potential and size distribution measurements were performed using Zetasizer Nano ZS automatic analyzer (Malvern Instruments, Malvern, UK) at 25 °C. A buffer at physiological pH was used to prepare the dispersions of nanoparticles for the dynamic light scattering (DLS) measurements. Prior to the DLS analysis, nanoparticles were dispersed in a buffer solution and sonicated for 5 min at room temperature. Samples were diluted several times to avoid aggregation and interaction of nanoparticles until stable data were obtained. The intensity, number, and volume size distributions were used for analysis. ζ-potential measurements were carried out using Smoluchowski model for analysis.

### 4.4. Electron Microscopy and UV-Vis Spectral Analysis

Cooper grids (200 mesh) with lacey carbon cover were treated with air plasma to make them hydrophilic. A total of 3 µL of the sample (L-ANP; H-ANP) was placed onto the hydrophilic grid in 100% humidity conditions and the excess of the sample was removed by blotting the grid for 1 s with filter paper. Then, the grid was immediately plunged into liquid ethane (automated plunging system, Vitrobot FEI, Waltham, MA, USA) and transferred in liquid nitrogen to cryo-TEM (transmission electron microscope Tecnai G 2 12 SPIRIT, FEI, USA). The morphology of the samples was characterized using scanning electron microscopy (SEM). SEM measurements were carried out using MERLIN (Carl Zeiss, Jena, Germany) with an acceleration voltage of 1 kV. For this, dried samples were coated with a gold thin film and imaged with SEM. UV-Vis spectra of samples were measured using a spectrophotometer (Shimadzu UV-3600 Plus, Kyoto, Japan).

### 4.5. Particle Loading and Release

Drug loading was performed by incubating 5 mg of particles in a solution of Sunitinib or Rhodamine 123 dissolved in DMSO at a concentration of 10 mg/mL or 5 mg/mL, respectively. The loading yield was calculated with the direct method, resuspending the particles in 10 mL of DMSO, and after keeping the solution overnight in agitation at 37 °C confirmed via the indirect method following the formula %EE = [(Drug added − Free “unentrapped drug”)/Drug added] ∗ 100. The concentration of the samples was evaluated via absorbance analysis at 420 nm of wavelength for Sunitinib or fluorescent analysis (Ex = 500; Em = 530) for Rhodamine 123 against a standard curve of known concentrations of these molecules. Release was performed in PBS using the same analytic approach.

### 4.6. Determination of Particle Degradation and Release in Proteolytic Conditions

The same amount of nanoparticles (2 mg) was resuspended overnight in 1 mL of different buffer solutions (Tris-HCl 0.05 M) under different temperature and pH conditions, with or without the presence of a proteolytic enzyme (1 mg/mL) as stated in the figure. All the 1.5 mL tubes were weighed before the experiment. The next day, the samples were centrifuged, the supernatant was discarded, and the tubes were dried overnight. The day after that, all the samples were weighed and the particle degradation was calculated as the difference of the weight between particles resuspended in a solution without and with the proteolytic enzyme. Similarly, a known amount of particles loaded with Sunitinib or Rhodamine 123 was incubated in trypsin solution for 1 h at 37 °C. The tubes were centrifuged and the supernatant collected. Sunitinib and Rhodamine 123 concentration was calculated via absorbance or fluorescence, respectively.

### 4.7. MTT Assay

To evaluate nanoparticle impact on cell viability, the cells were seeded in 96-well microplates (Costar, Corning Inc., Corning, NY, USA) at a density of 10 × 10^3^. Twenty-four hours after cell attachment, plates were washed with PBS, and the cells were treated with increasing concentrations, from 0.1 to 10 ng cell^−1^, of both L-ANP and H-ANP for 72 h. Six replicate wells were used for each control and tested concentration. The same procedure was applied to evaluate the toxicity of Sunitinib and particles loaded with this drug. In this case, the treatment was performed overnight, and then the cells were washed and the MTT analysis was performed after 48 h. The tetrazolium salt (MTT [3-(4,5-dimethylthiazol-2-yl)-2,5-diphenyltetrazolium bromide]) was dissolved in PBS (5 mg/mL) and added to 769-P and SKBR3 cells (100 μL mL^−1^ DMEM without serum or phenol red) according to the method of Mosmann [48]. After incubation for 3 h at 37 °C, a solution of 1 N hydrogen chloride–isopropanol (1:24, *v*:*v*) was pipetted into each well and mixed to dissolve the dark-blue formazan crystals formed. After a few minutes at room temperature, the plates were read at 570 nm in a BioTek Microplate reader (Winooski, VT, USA).

### 4.8. Flow Cytometry Analysis

Flow cytometry was used to evaluate amine saturation on the surface of the particles. In this case, L-ANPs and H-ANPs were labeled with Alexafluor 488 NHS ester following the instruction of the vendor and then analyzed with FACS. On the other hand, FACS was also used for evaluating the decrease of a fluorescent dye loaded in the particles after their internalization. In this case, both the particles were loaded with Rhodamine 123 and SKBR3 and 769-P cells were treated with L-ANPs and H-ANPs at a concentration of 1 ng cell^−1^ overnight. Then, the cells were washed and cultured as described before, fixed in 4% paraformaldehyde and resuspended in PBS at 4 °C. This procedure was repeated on different days after the treatment in a time window of 2 weeks. Analysis of cell fluorescence was performed on a BD FACSCanto II cytometer (BD Biosciences, Franklin Lakes, NJ, USA) with NovoExpress Software 1.4.1 (Agilent Technologies, Santa Clara, CA, USA) in the FITC-A channel; signals were plotted in linear mode. Percentage of GFP-positive cells was calculated compared to non-treated control cells.

### 4.9. Western Blot Analysis

SKBR3 and 769P were seeded at a confluency of 1 × 10^5^ cells cm^−2^. After 6 h of treatment with the particles, the culture medium containing NPs was removed. At 24, 48, and 72 h after the treatment, cells were washed twice with ice-cold PBS, were detached by gentle scraping, and were centrifuged at 250× *g* for 10 min at 4 °C. The cell pellets were lysed by using RIPA buffer (Thermo Scientific, Waltham, MA, USA) with 1 µL Halt protease inhibitor cocktail (Thermo Scientific) per 100 µL of buffer. A total of 30 µg of whole-cell protein extracts were loaded onto SDS/PAGE and then transferred on to a PVDF membrane for Western blot analysis, as previously described [8,43].

## 5. Conclusions

In conclusion, glutaraldehyde can increase drug loading and favor a more sustained drug release. The amount of cross-linker used has no significant impact on the physicochemical properties of the nanoparticles such as size and shape, but affects surface charge by saturating the amino groups of BSA. Glutaraldehyde also provides resistance to proteolytic degradation, determining particle drug delivery properties, but it does not affect the expression of the investigated lysosomal enzymes whose expression increased with both L-ANPs and H-ANPs. These data were confirmed by the higher cytotoxic properties observed in L-ANPs despite containing less therapeutic payload than H-ANPs.

Further studies will be necessary to unveil the effects of the cross-linker on particle porosity as a function of hydrophilic and hydrophobic payloads and to reveal the mechanistic involvement of the amine groups in particle degradation.

In this scenario, the ANP model and specific chemical surface modifications represent optimal tools to investigate cellular proteolytic activity and protease expression, shedding light on the lysosomal degradome homeostasis that comprises numerous members and natural inhibitors.

## Data Availability

Not applicable.

## Supplementary Materials

The following supporting information can be downloaded at: https://www.mdpi.com/article/10.3390/ijms241210245/s1.
