# Supplementary material for: Proteolytic Resistance Determines Albumin Nanoparticle Drug Delivery Properties and Increases Cathepsin B, D, and G Expression
Source: Int J Mol Sci. 2023 Jun 16;24(12):10245. doi: 10.3390/ijms241210245 (PMC10299664; doi:10.3390/ijms241210245)
Supplement: Supplementary file 1 [file ijms-24-10245-s001.zip › ijms-2427987-supplementary.pdf]

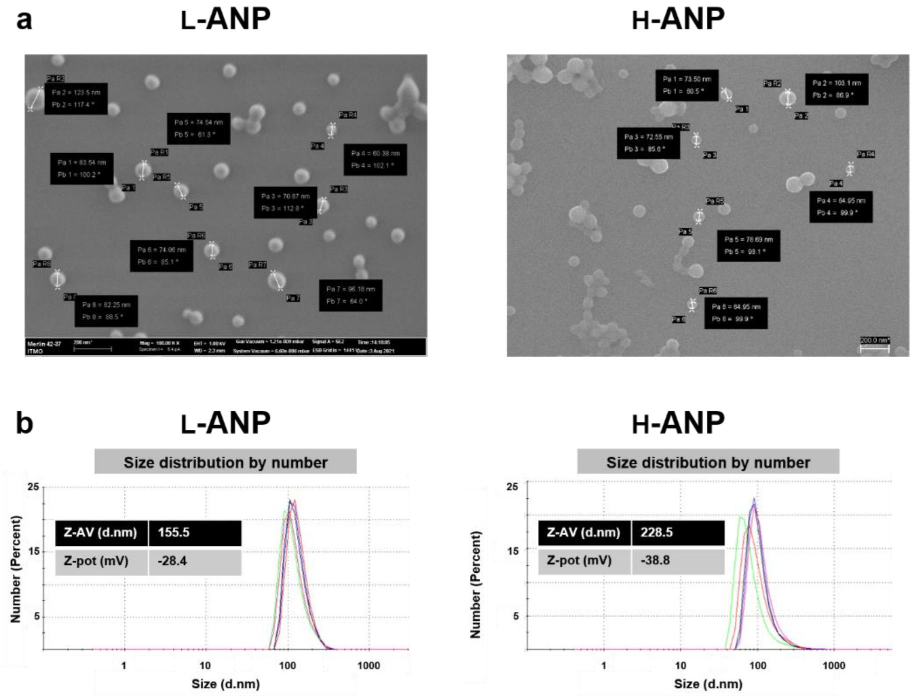

Figure S1 Physical and chemical properties of the particles: (a) Particle SEM analysis, scale bars correspond to 200 nm, particle size analysis is shown. (b) DLS analysis and Z-pot measurement in the table

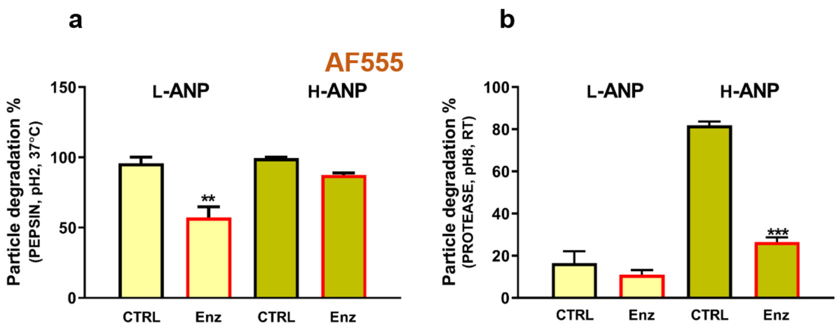

Figure S2 Nanoparticle resistance vs proteolytic activity: (a) Nanoparticle degradation after incubation with Pepsin. Albumin was previously labelled with AF555 (b) Nanoparticle degradation after incubation with commercial protease.

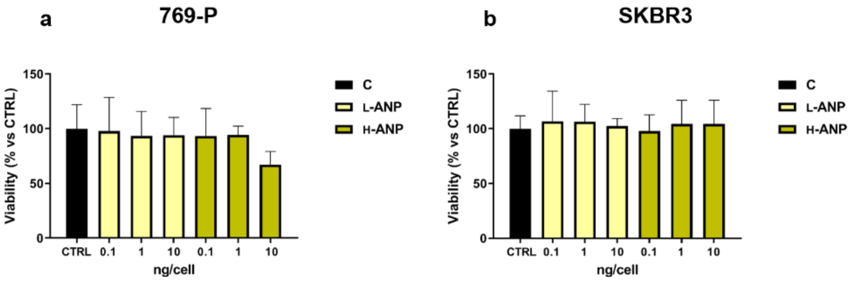

Figure S3 ANP impact on cell viability: (**a**) Cell viability of 769-P and (**b**) SKBR3 after incubation for 72 hours with increasing concentrations of L-ANP and H-ANP.
